# Supplementary material for: Transferrin-Enabled Blood–Brain Barrier Crossing Manganese-Based Nanozyme for Rebalancing the Reactive Oxygen Species Level in Ischemic Stroke
Source: Pharmaceutics. 2022 May 25;14(6):1122. doi: 10.3390/pharmaceutics14061122 (PMC9231148; doi:10.3390/pharmaceutics14061122)
Supplement: Supplementary file 1 [file pharmaceutics-14-01122-s001.zip › pharmaceutics-1680444-supplementary.pdf]

Supplementary File

Transferrin-Enabled Blood–Brain Barrier Crossing Manganese-Based Nanozyme for Rebalancing the Reactive Oxygen Species Level in Ischemic Stroke

Qianqian Zhao [1,†], Wenxian Du [1,†], Lingling Zhou [1], Jianrong Wu [2], Xiaoxing Zhang [1], Xiaoer Wei [1], Sijia Wang [1], Yu Huang [1] and Yuehua Li [1,*]

[1] Department of Radiology, Shanghai Jiao Tong University Affiliated Sixth People's Hospital, Shanghai Jiao Tong University School of Medicine, 600 Yi Shan Road, Shanghai 200233, China; zhaoqianqian199010@gmail.com (Q.Z.); wx0910@mail.ustc.edu.cn (W.D.); llzhou21@m.fudan.edu.cn (L.Z.); dr.zhang8967@gmail.com (X.Z.); weixiaoer13774356395@gmail.com (X.W.); sjwang121@gmail.com (S.W.); yuhuang6y@163.com (Y.H.)

[2] Department of Ultrasound in Medicine, Shanghai Jiao Tong University Affiliated Sixth People's Hospital, Shanghai Jiao Tong University School of Medicine, 600 Yi Shan Road, Shanghai 200233, China; wujr_028@126.com

* Correspondence: liyuehua77@sjtu.edu.cn

† These authors contributed equally to this work.

Experimental

Drug release by EMT in vitro

In order to evaluate drug release by EMT, four experimental groups (control, Eda, MT and EMT) were established. Similarly, the EMT group was subdivided into groups with concentrations of 12, 25, 50, 100 ppm. Then, 10 mg/L EMT was added to PBS buffer solution (20 mL) at pH 7.4, 6.4, or 5.0. The above mixed solutions were added to dialysis bags, then placed in a flask containing new PBS on a thermostatic culture oscillator (sky-100c) operating at 800 rpm. Aliquots (2 mL) of the solutions were sampled at specific time points, and the drug release of Eda was evaluated by UV-vis analysis.

In vitro imaging

A 3.0T MRI system (Prisma, Magnetom, Germany) was used for in vitro T1 weighted MRI. The EMT was dispersed in PBS at different pHs with or without H_2O_2 at different concentrations. The longitudinal relaxation rates (r1) for EMT in PBS were determined at pH 7.4, 6.4, and 5.0, and $[H_2O_2]$ = 400, 200, 100 μM. Different manganese

concentrations were also used (0.2, 0.1, 0.075, 0.05, and 0.025 mM) . The test parameters were as follows: tr = 8000 MS, TE = 1 ms, RfD = 0.2 Ms.

Cell experiments

Antioxidant protection in vitro

HUVECs were cultured, digested, and counted as above. Cells were grown in 96-well culture medium plates at a density of 10^5 cells/well for 24 h. Then, a solution of H_2O_2 (400 μM) was added to each well, incubating with HUVEC cells for 0, 0.5, 1, 2 and 4h, respectively. Then cells were washed with PBS, and new media EMT (100 ppm) was added to the wells for 6 h. Cell viability was measured by CCK-8 assay. According to the results above, we further cultivated cells in 96-well and added H_2O_2 (400 μM) into wells, incubating about 2h. The HUVECs were washed with PBS, and new media containing Eda, MT, or EMT (50 ppm) was added to the wells. We also performed the experiments with EMT at different concentrations (0, 12.5, 25, 50, 100 ppm). After treatment for 6 h, CCK-8 assays were performed to assess cell viability.

Cell uptake

The EMT nanozyme was mixed with 0.5 mg FITC in 5 mL water and stirred overnight. The mixture was then dialyzed in DI water using dialysis bag with a MWCO of 3.5 kDa and with the water being changed every 6 h. Furthermore, HUVECs were seeded into 6-well microplates at a density of 10^5 cells/well. Following the addition of EMT (50 ppm) dispersed in DMEM culture medium and incubation for 1, 2, 4, or 8 h, the nanozyme was removed by flushing with PBS three times. Finally, the cells were harvested to perform quantitative analysis of cell uptake by flow cytometry (BD LSRFortessa).

BBB in vitro model

Bend.3 cells were seeded on 24-well trans-well filters with a membrane of 1.0 mm thickness (FALCON Cell Culture Insert, Becton Dickinson Labware, USA) for 7-14 days. Once the cells reached 90% confluence, an epithelial voltohmmeter (Millicell-RES,

Millipore, USA) was used to measure transepithelial electrical resistance (TEER) values. When the values reached 200 Ω, they were selected to perform the testing. Free EMT nanozyme (50 ppm) were added to the apical chamber of the model with 50 rpm shaking at 37 °C overnight to determine the transport profile. Then, the Mn concentrations in the apical lateral chamber, the filter membrane, and the basolateral medium were determined by ICP-OES. Finally, to further investigate the Tf-mediated mechanism, we carried out another control study in parallel by adding free Tf with a dose of 3 mg/mL to the upper lateral chamber 1 h before adding EMT nanozyme. Then, the above mentioned steps were repeated.

Fluorescence microscope imaging of ROS in cells

The cells were incubated in confocal laser scanning microscope (CLSM) culture dishes for 12 h at a cell density of 5×10^3 per sample. The following five experimental groups were established: no treatment (control group), H_2O_2, Eda + H_2O_2, MT + H_2O_2, and EMT + H_2O_2. After 4 h of co-culture with different nanozyme (100 μg/mL), DCFH-DA was added, and the samples were observed with CLSM after 1 h. Image Pro Plus was used to determine fluorescence intensity.

Quantitative level of intracellular ROS by flow cytometry results

The cells were incubated in 6-well plates at a density of 5×10^3 per well for 12 h. Then, H_2O_2 (400 μM) was added to the wells, incubating for 1 hour. After removing and dishing, the H_2O_2, Eda, MT (50 ppm), and EMT (50 ppm) were dispersed in DMEM and co-incubated with the cells for 4 h respectively. To evaluation of ROS-removal effect at cell level, we added DCFH-DA into cells, incubating for 20 minutes. The results of cell staining were observed by flow cytometry. We performed the experiments with EMT at different concentrations (0, 25, 50, 100 ppm) and H_2O_2 at a concentration of 400 μM.

Animal experiments

Fluorescence imaging of rats

The animal model of ischemic stroke was established by occlusion of the middle cerebral artery (MCAO) in rats with traditional thread occlusion method. The EMT nanozyme was mixed with 0.5 mg Cy5.5 in 5 mL water and stirred overnight. The mixture was then dialyzed in DI water using dialysis bag with a MWCO of 3.5 kDa and with the water being changed every 6 h. For sham group was injected with Cy5.5. In TfR-blocked group, Holo-Tf solution (dissolved in saline, 10 mg kg^{-1}, 1 mL) was i.v. injected 12 h before the injection of Cy5.5-EMT to saturate the TfR. In third group, EMT labeled with Cy5.5 (Cy5.5-EMT) was injected into MCAO rats via caudal vein with a concentration of 0.136 mmol Mn kg^{-1}. Images were collected at 0, 1, 2, 3, 4, 5, 6, 8 and 12 hours after Cy 5.5-EMT injection. After the probe was injected into the body, the stroke rats were killed with brain, heart, liver, spleen, lung, kidney stripped, and exposing them to the same parameters used for fluorescence imaging. The fluorescence signals and images of the whole body and brain of SD rats were obtained by Bruker (Billerica, Ma, USA) imaging system. The excitation light was set at 673 nm and the receiving light was set at 707 nm.

Objective to evaluate the effect of antioxidation in vivo on brain protection

In order to study the neuroprotective effect of EMT, the experimental animals were randomly divided into three groups. 1) Sham operation group (saline + sham group): MCAO sham operation was performed, that is, only the skin of the neck was cut, but no arterial embolization was performed, 2) Saline ischemic stroke model group (saline + MCAO group): MCAO operation, 90 min after reperfusion, saline injection via tail vein; 3) IS model group (EMT + MCAO group): EMT nanozyme were given via tail vein for 1 day. After MCAO and 1.5 hours of reperfusion, saline and EMT particles were injected into the cerebral apoplexy model rats via tail vein. 24 h later, the brain was decapitated, frozen at – 20 °C for 20 min, made into coronal slices, about 2 mm thick, then stained with 1% TTC solution at 37 °C. Finally, the stained brain tissue was photographed by digital camera, and the volume percentage of damaged brain tissue

was calculated by Image Pro Plus analysis, so as to obtain the best therapeutic dose. At the same time, in order to compare the therapeutic effect of Eda, MT and EMT nanozyme, the same dose of nanozyme were injected into different groups of MCAO rats via tail vein. The brain slices were fixed with 10% formaldehyde. The normal brain tissue was red, while the infarct area was pale white.

ELISA assays

Rats were divide into groups just as the TTC assays, including sham operation group (saline + sham group), saline ischemic stroke model group (saline + MCAO group), AIS model group (Eda, MT, EMT + MCAO group) with the concentrations of 0.6mg/kg. After reperfusion, nanoparticles were injected into rats via tail vein. 24 h later, the brains were harvested, smashed, from which protein was extracted. Then we used mouse TNF-α, IL-1β and IL-6 ELISA kits (Anogen) to test the level after treatment.

Evaluation of alleviating the damage of BBB

After 22 h of cerebral ischemia, the rats were injected with the same amount of normal saline, Eda, MT and EMT (0.8 mg/kg) via tail vein. After that, EB, a tracer of BBB permeability, was dissolved in normal saline and injected into the above rats and healthy rats (2%, 4 mL/kg) via tail vein. Circulating for 2 h, with the chest wall of rats opened and the left ventricle perfused with normal saline, and auricula dextra was cut apart. When the clear saline flow out from auricula dextra, formaldehyde was injected into left ventricle. Then the brain was decapitated, weighed and photographed with digital camera. At the same time, the brain tissue was frozen and cut into 10 μm thick slices. The fluorescence signal of EB in brain tissue was detected by CLSM at 620 nm excitation wavelength.

Detection of ROS in vivo

To further prove the antioxidant mechanism of EMT nanozyme in protecting brain tissue in vivo, DHE-ROS assay kit (BB-47051, BestBio) was used according to the standard protocol. Then the frozen section of brain tissue (10 μm) in MCAO group and

MCAO +EMT group were stained with DCFH-DA fluorescent probe at the concentration of 1:500.

Supplementary figures:

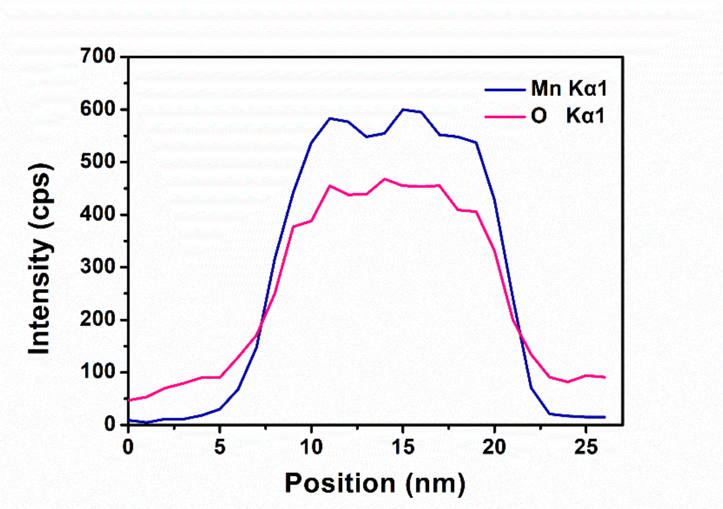

Figure S1. The EDS element line scanning scope. The blue curve shows Mn signal intensity while the red curve shows O signal intensity.

Table S1. Peak Fitting of O 1s spectrum of EMT.

**Peak**	**State**	**B.E. (eV)**	**FWHM (eV)**	**Area**	**Atom %**
O1	adsorbed oxygen	533.5	1.72	2135.9	21.7
O2	oxygen defect	532.0	1.72	7262.2	73.5
O3	lattice oxygen	530.4	1.54	472.4	4.8

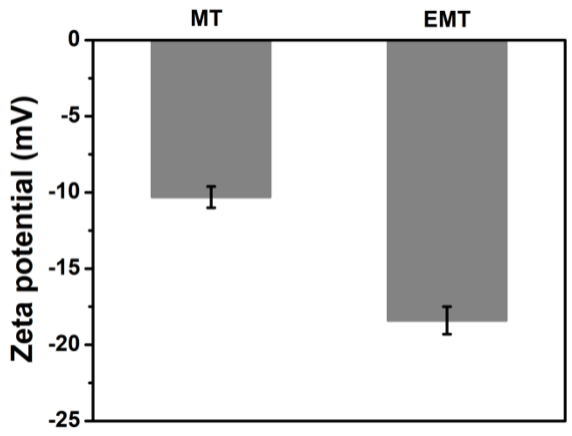

Figure S2. Zeta analysis of MT and EMT nanozyme.

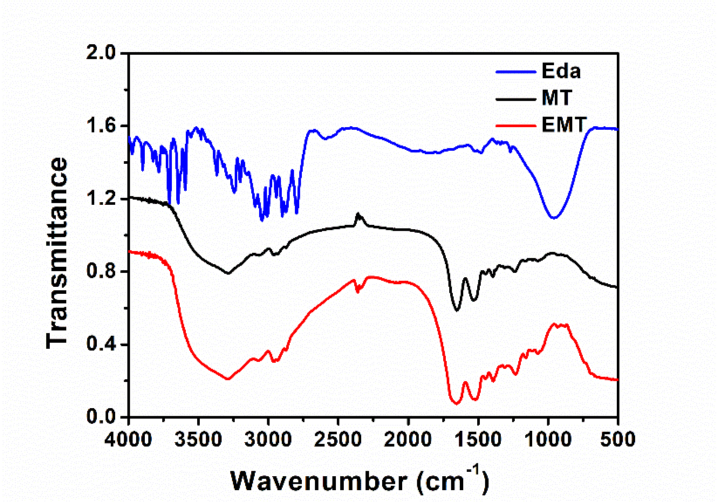

Figure S3. FTIR spectra of MT and EMT nanozyme.

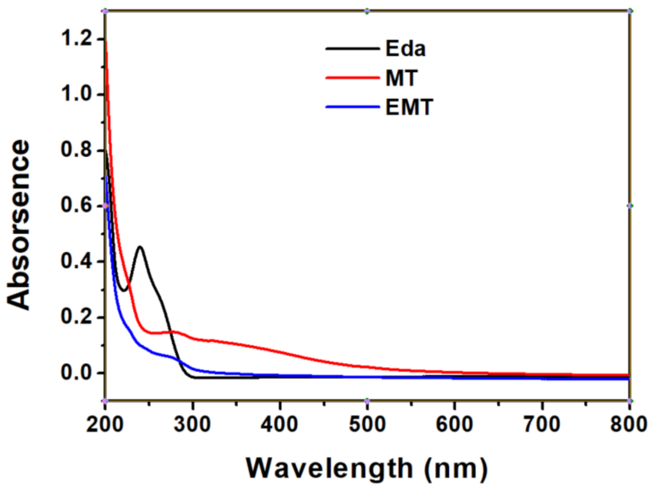

Figure S4. UV–vis absorption spectra of Eda, MT and EMT.

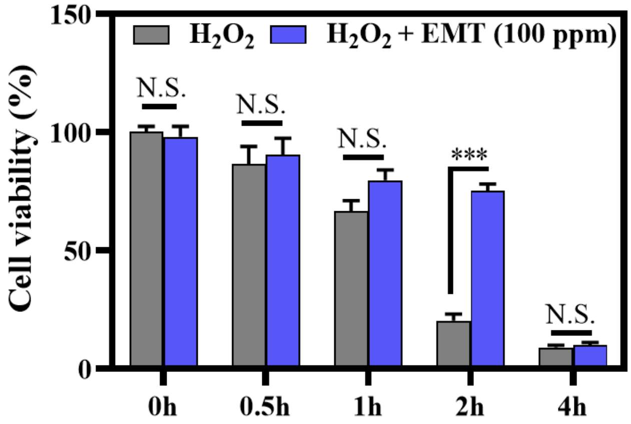

Figure S5. Cell viability of incubating with H_2O_2 (400 μM) for 0 h, 0.5 h, 1 h, 2 h and 4h, respectively and the treatment effect of the EMT nanozyme at a concentration of 100 ppm for 6 h. *** $p < 0.001$.

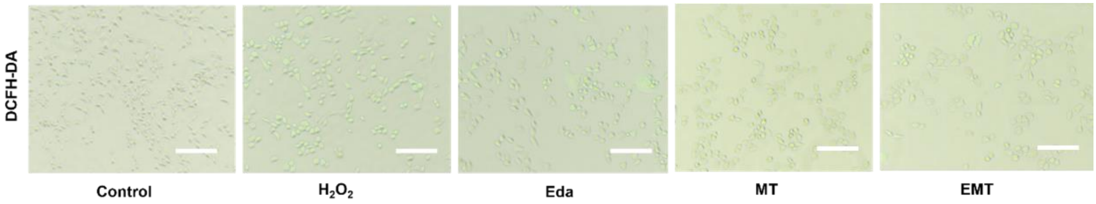

Figure S6. DCFH assay results were used to evaluate ROS-relieving ability. Scale bar, 100 μm.

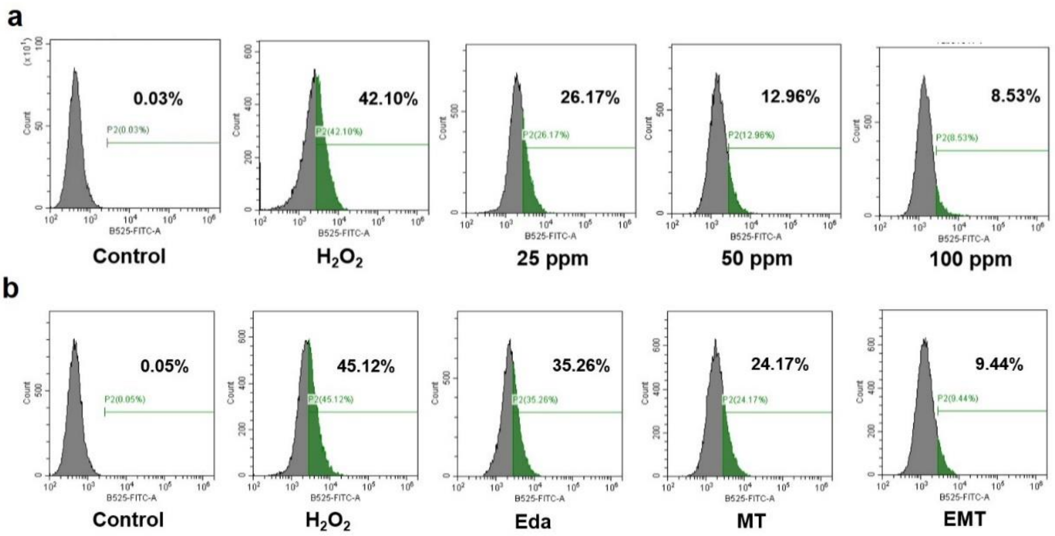

Figure S7. Flow cytometry data for showing intracellular level of total ROS after treated with different groups including (a) Control, H_2O_2, 25 ppm, 50 ppm, and 100 ppm. and (b) Control, H_2O_2, Eda, MT, and EMT.

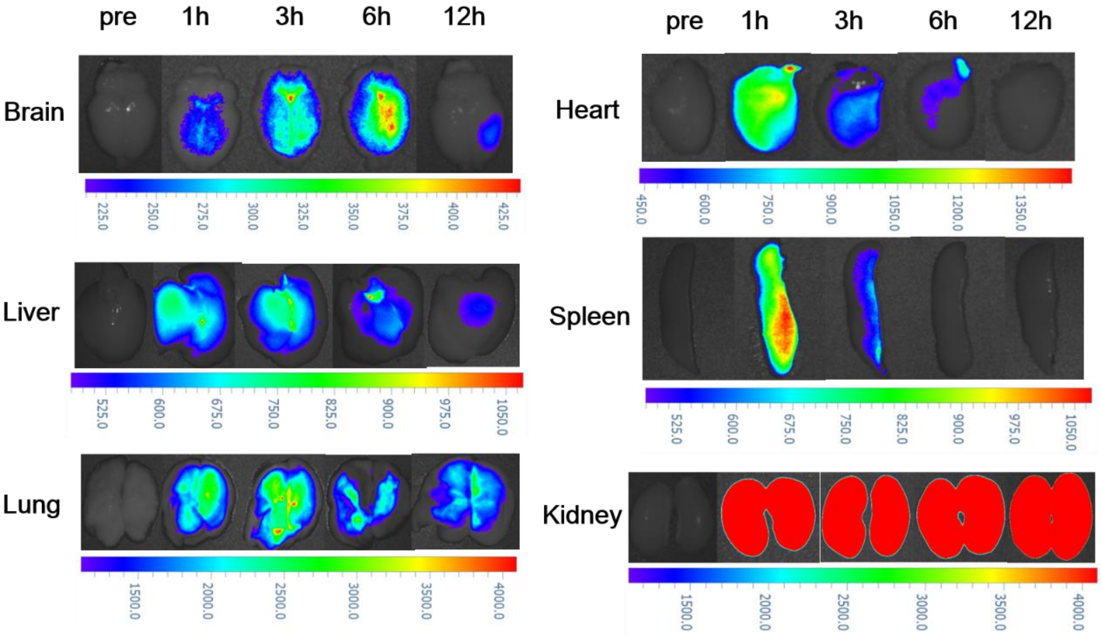

Figure S8. Fluorescence imaging of the excised major organs of rats before and after injection with cy5.5-labeled EMT over time (1, 3, 6, and 12 h).

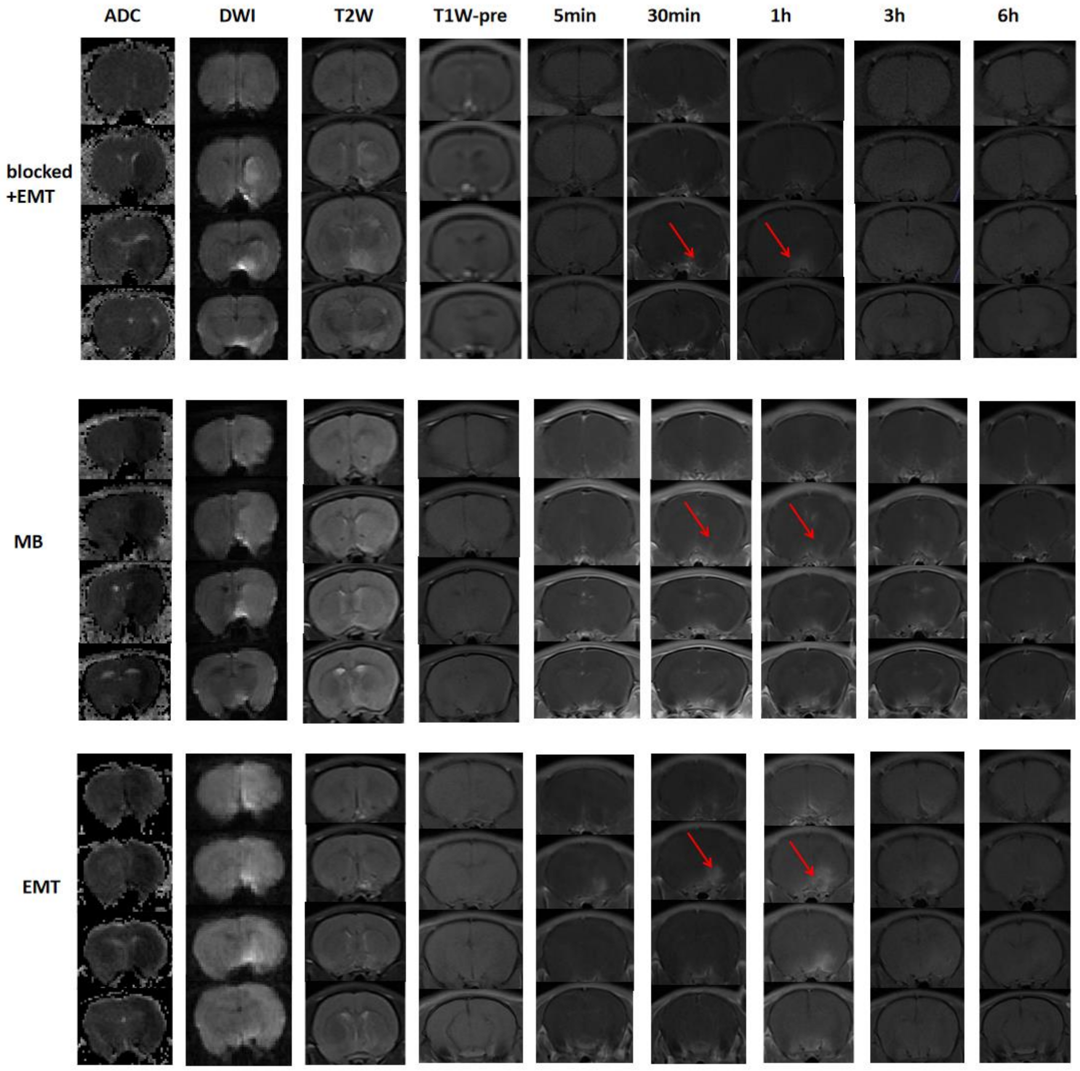

Figure S9. MRI imaging in MCAO rats. MR signal variations (DWI, ADC, T_2, T_1, T_1-enhanced scanning) of the ischemic areas for different groups. Red arrows indicate the location of the infarct area.

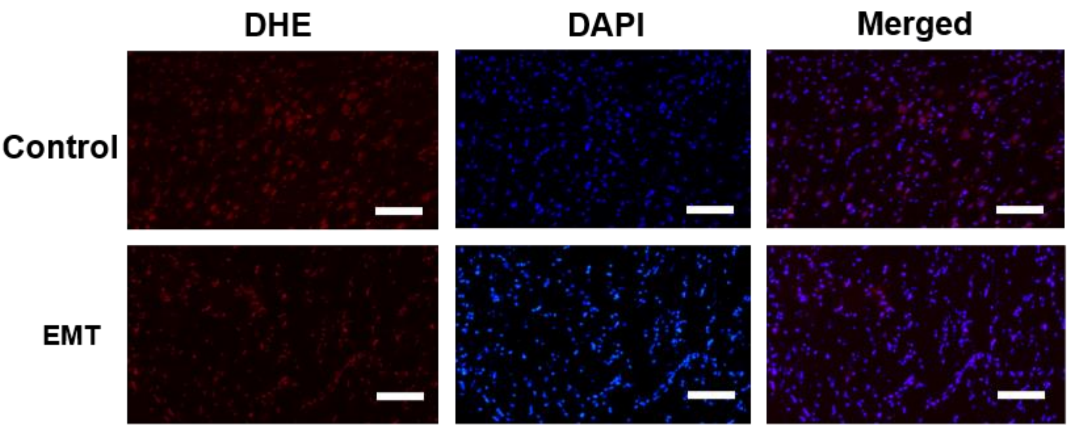

Figure S10. Confocal images of red fluorescence signals from DCF used to detect ROS levels in ischemic brain tissue. Scale bar, 50 μm.

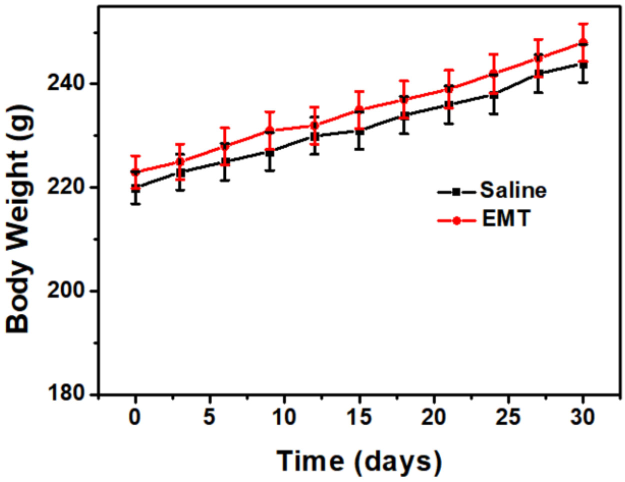

Figure S11. The body weight changing curves of SD rats in different groups after treatments.

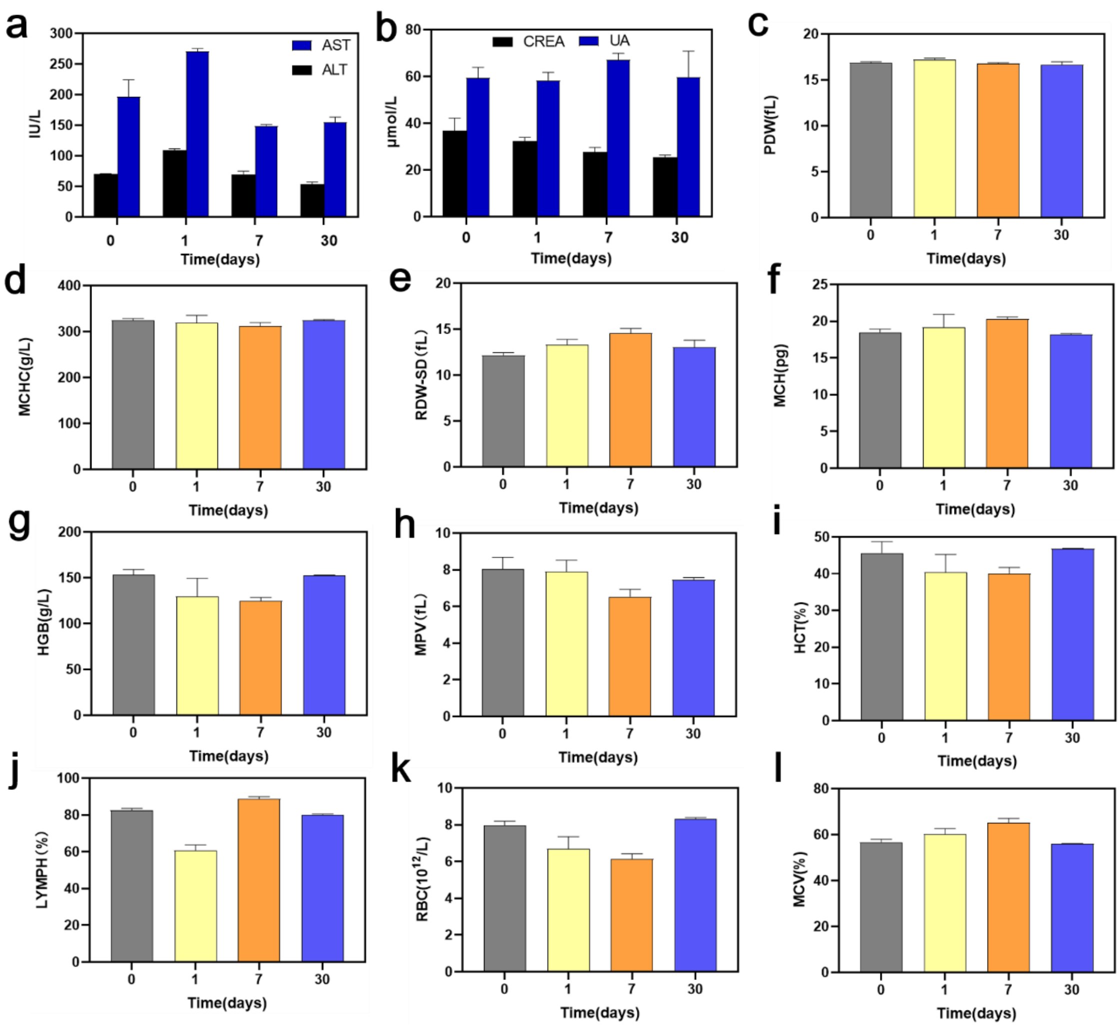

Figure S12. Variations of different blood indexes in rats from the saline control group and groups treated with EMT nanozyme at a dose of 10 mg/kg after 1, 7, and 30 d of normal feeding. The key biochemistry parameters(a–b), like hepatic and renal function (ALT, AST, CREA, UA) and the different vital hematology markers (c–l), including PDW, MCHC, RDW-SD, MCH, HGB, MPV, HCT, LYMPH, RBC and MCV, fluctuated within their normal ranges, indicating that EMT had negligible toxicity.
